# A New Lysosome-Targeted NIR Fluorescent Probe for Specific Detection of Cysteine over Homocysteine and Glutathione

**DOI:** 10.3390/molecules28176189

**Published:** 2023-08-22

**Authors:** Qiuchen Liu, Chang Liu, Song He, Xianshun Zeng, Jian Zhang, Jin Gong

**Affiliations:** 1 School of Pharmacy, Weifang Medical University, Weifang 261053, China; 2 Tianjin Key Laboratory for Photoelectric Materials and Devices, School of Materials Science & Engineering, Tianjin University of Technology, Tianjin 300384, China; 3 School of Chemical Engineering and Technology, Tianjin University, Tianjin 300072, China

**Keywords:** cysteine, near-infrared, fluorescent probe, cell imaging, lysosomal targeting

## Abstract

In this paper, by modifying the thioxanthene-benzothiozolium fluorophore, BCy-Cys, a lysosome-targeted near-infrared (NIR) fluorescent probe was synthesized for the detection of cysteine (Cys) from homocysteine (Hcy)/glutathione (GSH). As expected, BCy-Cys exhibited high selectivity and high sensitivity for detection of Cys over Hcy/GSH, with an extremely low limit of detection at 0.31 μM, marked by obvious color changes. HRMS was conducted to confirm that the fluorescence intensity at 795 nm was significantly enhanced by the enhancement of intramolecular charge transfer (ICT). Importantly, BCy-Cys could be used to visualize both exogenous and endogenous lysosomal Cys, signifying its potential application in complex organismal systems.

## 1. Introduction

Cysteine (Cys), homocysteine (Hcy), and glutathione (GSH) are very important biothiols in organisms, which play an indispensable role in signal transduction and biocatalysis of physiological processes and biological systems [1,2,3], in particular, maintaining intracellular oxidation-reduction equilibrium and higher-order structures of proteins by utilizing the balance between thiol (RSH) and disulfide (RSSR) [4]. Studies have shown that Cys, Hcy, and GSH were structurally similar and interrelated in biological systems, but their specific physiological roles were different, and abnormal levels could lead to different diseases [3,5]. As a proprotein amino acid, Cys (30–200 μM) was a source of sulfides in the body’s metabolism and a metabolite of Hcy, which was essential for the synthesis of peptides, proteins, acetyl-CoA, and antioxidant glutathione [6,7,8]. Abnormal levels of Cys could lead to associated health conditions, for example, skin disruption, hair desaturations, slow growth, liver dysfunction, edema, debilitation, severe circulatory disorders, and neutoxicity [9,10]. In particular, it is suspected that Cys can degrade intralysosomal Cys-containing proteins by reductive cleavage of disulfide bonds [11,12], and participate in increasing the rate and magnitude of thyroidal protease reactions with thyroglobulin [13]. Hcy is an important intermediate product of methionine metabolism and is one of the culprits in stroke and Alzheimer’s disease [5]. Many scientists consider elevated homocysteine to be an important risk factor for cardiovascular disease and refer to it as the “cholesterol of the modern world” as a new early warning indicator. GSH is composed of glutamic acid, cysteine, and glycine, which acts as an important antioxidant in the body, preventing damage to important cellular components by reactive oxygen species such as free radicals, peroxides, lipid peroxides, and heavy metals and protecting the sulfhydryl groups in many proteins, enzymes, and other molecules. Many common current diseases have been linked to excessive depletion of glutathione, including but not limited to neuropathy, cardiovascular disease, chronic age-related diseases, diabetes, and accelerated aging [3]. Therefore, the development of reliable and valid bio-analytical methods for detecting Cys with high selectivity over Hcy/GSH was of great value, especially for the sensing and imaging of Cys in the lysosome of living cells.

In recent years, with the development of fluorescent probe technology, real-time monitoring of biothiols has become feasible. Its advantages include high selectivity, high sensitivity, non-invasiveness, and the capability for real-time sensing in biological systems [14]. For instance, Guo et al. described a coumarin-hemicyanine fluorescence probe monitoring intracellular Cys and GSH [15]. The probe can utilize different reaction sites to interact with Cys, GSH, and Hcy to generate different optical signals to differentiate between them, accompanied by excellent optophysical properties and non-destructive imaging ability. Thus far, the reported Cys probes were mostly designed based on the high nucleophilicity of sulfur atoms, including aldehyde cyclization [16], cleavage reactions [17], 1,2-addition reactions [18], and aromatic substitution-rearrangement cascade reactions [19]. However, few lysosome-targeted fluorescent probes for the detection of Cys have yet been successfully established [20,21], and few probes could be used to distinguish Cys well from Hcy/GSH due to their highly homogeneous structure and chemical reaction activities [20,21], although most probes could highly selectively distinguish these biothiols from other amino acids. They all contain amino, carboxyl, and sulfhydryl groups. The amino groups are all on the ortho-carbon atoms of the carboxyl group. The difference is that their molecular sizes are different, with GSH greater than Hcy, which is greater than Cys, and the distance between the sulfhydryl group and the amino group being different [1,2,3]. For example, Zeng et al. described a lysosome-targeting sensor for recognizing Cys from other biothiols [22]. The probe was able to efficiently and specifically identify Cys in lysosomes even in the presence of thiols and other amino acids, and the emission wavelength of the probe could reach the near-infrared region. Meanwhile, biological reports indicate that probes with near-infrared (NIR) emission can increase their ability to penetrate tissues [23]. As a consequence, designing and developing specific probes to detect and accurately evaluate Cys levels in lysosomes remains highly desirable.

To address these issues, our group has spared no effort in attacking the challenges of lysosome-targeted probes with near-infrared (NIR) emission and has made some progress in this field, which provides the basis and inspiration for us to continue to delve deeper into the research and problem solving [24,25]. In this paper, based on previous research on lysosomal targeting [25], a lysosome-targeted NIR fluorescent probe, BCy-Cys, was designed and fabricated to discriminate Cys from Hcy/GSH by modifying the thioxanthene-benzothiozolium fluorophore and acryloyl group. By exploiting the differences in the nucleophilic attack of thiols toward the double bond and steric hindrance, BCy-Cys can distinguish Cys from Hcy/GSH. Unsurprisingly, the probe BCy-Cys was not only able to demonstrate the emission wavelengths in the NIR region but also nondestructively detected Cys within the lysosome, better realizing our design intent. The probe is capable of emitting at a wavelength of 795 nm after Cys treatment, which is a very high wavelength. More specifically, BCy-Cys exhibited high selectivity and high sensitivity for the rapid detection of Cys over Hcy/GSH, with an extremely low limit of detection at 0.31 μM. Furthermore, it is also noteworthy that the color of the solution turned from bright blue to cyan in the presence of Cys, suggesting that BCy-Cys can be used for ‘naked eye’ determination of Cys. Compared with the reported probes, BCy-Cys was able to better discriminate Cys from Hcy/GSH and showed a lower limit of detection and a faster response time (Table S1). Importantly, BCy-Cys can be accumulated in the lysosome and used for monitoring endogenous lysosome Cys in real-time. 

## 2. Results and Discussion

**Scheme 1:**
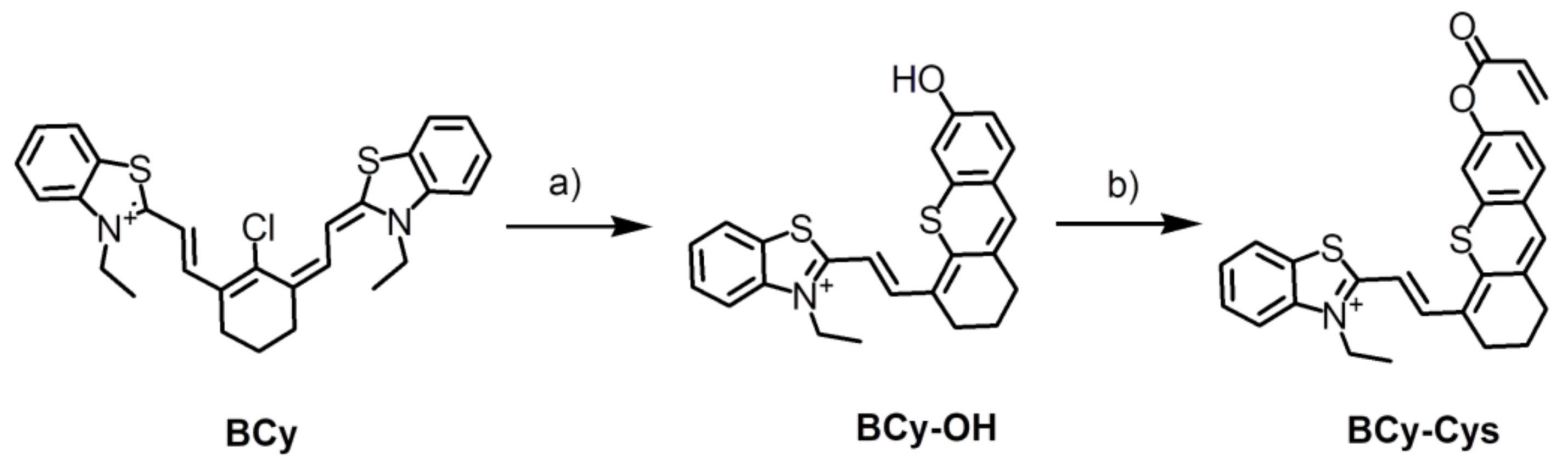
Synthesis of BCy-Cys. (**a**) Resorcinol, anhydrous potassium carbonate, acetonitrile, 60 °C, 5 h, 61%; (**b**) acryloyl chloride, Et_3_N, dichloromethane, 0 °C to room temperature, 4 h, 20%.

The probe BCy-Cys was synthesized according to Scheme 1. There was no significant difference in the yield of BCy, the key ingredient, which was synthesized via a two-step process in 61% yield, in keeping with the literature [24]. The detailed reaction reagents and conditions are depicted in Scheme 1. At the beginning, key compounds BCy and resorcinol were subjected to a similar retro-Knoevenagel reaction to produce the blue powder compound BCy-OH in a high yield of 61%. With triethylamine as the acid binding agent in dichloromethane, followed by treatment by acryloyl chloride and the compound BCy-OH, the probe BCy-Cys was produced in a 20% yield. NMR and HRMS were used to characterize and confirm the structures of BCy-OH and BCy-Cys, and the specific data could be found in the supporting information (Figures S7–S12).

**Figure 1:**
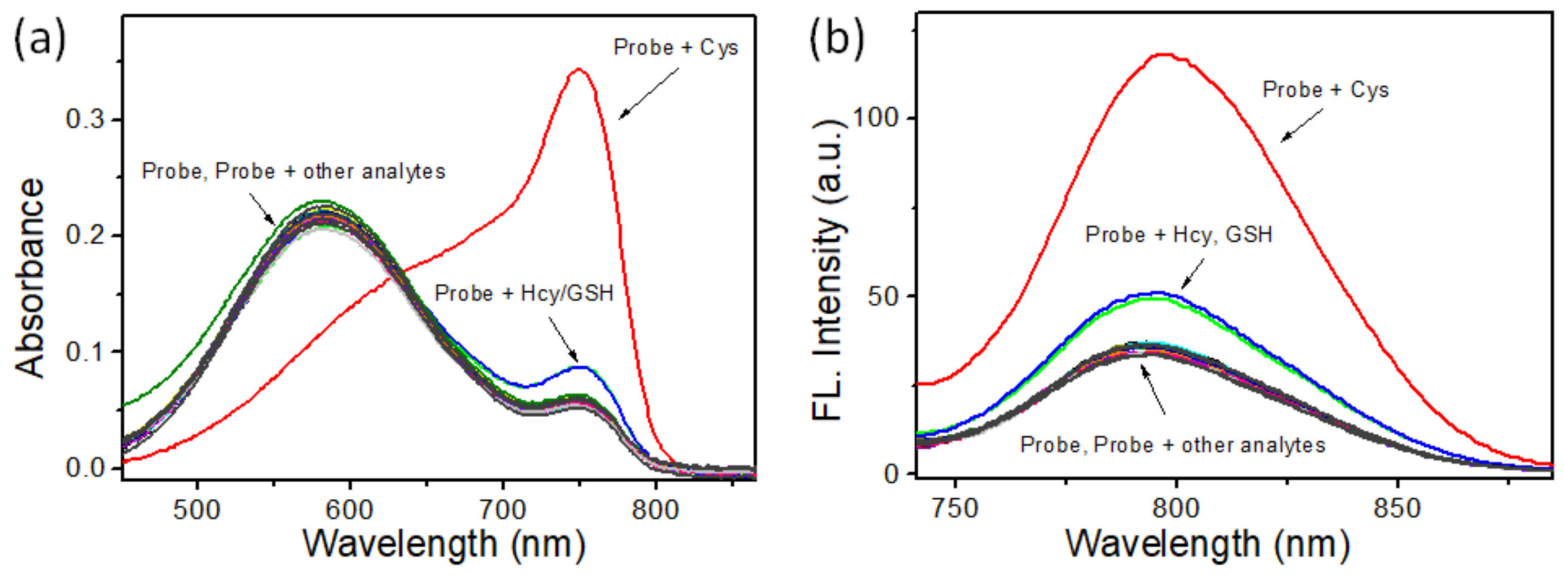
(**a**) UV-visible absorption spectra and (**b**) emission spectra of BCy-Cys (10 μM) after 15 min of reaction with the addition of various different analytes (100 μM). Various analytes: (1) Phe, (2) Ala, (3) Met, (4) Gln, (5) Arg, (6) Cys, (7) Lys, (8) Tyr, (9) Leu, (10) Pro, (11) Trp, (12) Ser, (13) Thr, (14) Val, (15) His, (16) Hcy, (17) GSH (1 mM), (18) Na^+^ (1 mM), (19) K^+^ (1 mM), (20) Ca^2+^ (1 mM), (21) Mg^2+^ (1 mM). The standard: PBS buffer solution (10 mM, pH 7.4, 25% EtOH), λ_ex_ = 720 nm, slit = 5/5 nm.

With the probe BCy-Cys in hand, we first investigated the capability of BCy-Cys as an activatable fluorescent probe toward Cys under physiological conditions (PBS, 10 mM, pH 7.4, 25% ethanol, and room temperature). For assessing the specific nature of BCy-Cys for Cys, the probe BCy-Cys was separately incubated with various amino acids and biothiols (Phe, Ala, Met, Glu, Arg, Cys, Lys, Tyr, Leu, Pro, Trp, Ser, Thr, Val, His, GSH, and Hcy) and metal ions (Na^+^, K^+^, Ca^2+^, and Mg^2+^) under the same experimental conditions. As shown in Figure 1a, upon treatment with Cys (100 μM), the maximum UV-visible absorption band of BCy-Cys at 585 nm vanished, while a new peak appeared at 750 nm. In contrast, there is negligible signal change in response to the other amino acids, the biothiols Na^+^, K^+^, Ca^2+^, and Mg^2+^. Meanwhile, we found that the addition of Cys (100 μM) to a solution of BCy-Cys dramatically intensified the fluorescence band at 795 nm (Figure 1b). In contrast, under identical conditions, only negligible fluorescence changes were observed at 795 nm after the addition of other analytes. Surprisingly, upon treatment with Hcy (100 μM) and GSH (1 mM), both absorption and fluorescence spectra showed negligible changes. It is also noteworthy that the color of the solution turned from bright blue to cyan in the presence of Cys (Figure S1), suggesting that BCy-Cys can be used for ‘naked eye’ determination of Cys. These phenomena demonstrated that BCy-Cys can positively recognize Cys with higher specificity than other substances, especially Hcy and GSH. 

In addition, to further assay the specific identification of BCy-Cys to Cys, the tests were also conducted with the incorporation of Cys into the solution of BCy-Cys in the presence of the other analytes above-mentioned, respectively. As illustrated in Figure S2, the co-existence of relevant amino acids, biothiols, and metal ions barely interfered with the discriminatory characterization of BCy-Cys. These inspiring results suggested that BCy-Cys was highly selective for Cys over the other biologically relevant analytes, even in the presence of GSH and Hcy, demonstrating the potential for detecting Cys under complex physiological conditions.

**Scheme 2:**
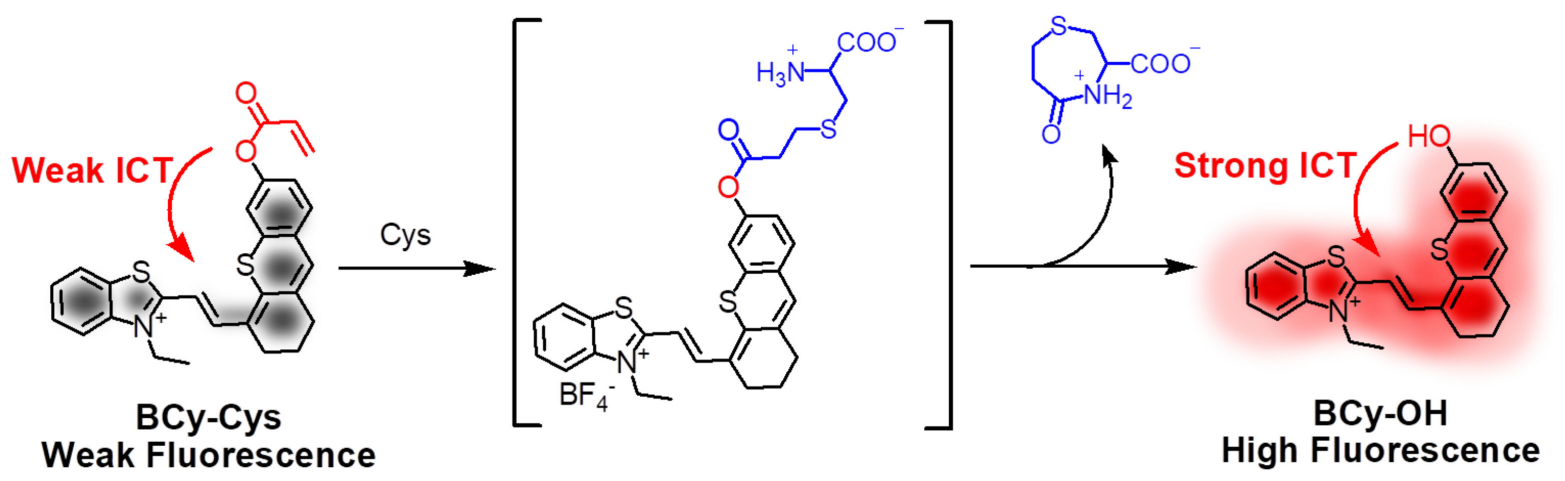
The hypothesized mechanism by which BCy-Cys characterized the release of strong fluorescent signals from Cys.

According to the aforementioned speculation and the reported mechanism [18], we assume that the above-mentioned spectral and color changes are caused by the enhancement of ICT. As far as we know, the acrylic group, as a strong electron-withdrawing group, restricts ICT [18], leading to quenching of BCy-Cys fluorescence. The -OH group is an electron-releasing group in the free state, which releases electrons to produce ICT, and when it is blocked by acryloyl, which is an electron-absorbing group, it is clear that electron transfer is difficult. However, when the -OH group is released in the presence of Cys, the ICT is enhanced, leading to the recovery of fluorescence (Scheme 2). Furthermore, the reaction mechanism was explained by HRMS as follows: The HRMS was subsequently undertaken to quantify and criticize the molecular weight of the probe BCy-Cys after the reaction with Cys. As depicted in Figure S3, a prominent peak at *m/z* 404.1143 corresponding to [BCy-OH]^+^ (calcd. 404.1143 for C_24_H_22_NOS_2_^+^) was clearly observed in the HRMS report, which provided evidence for the assumption above-mentioned. In addition, the ultraviolet-visible absorption and fluorescence emission spectra of BCy-Cys (10 μM) were measured after Cys (100 μM) treatment in PBS buffer (25% ethanol) and then compared with the spectra of BCy-OH (10 μM). As shown in Figure S4, the ultraviolet-visible absorption spectra of BCy-Cys treatment with Cys and the ultraviolet-visible absorption spectra of BCy-OH overlap well, implying that BCy-Cys undergoes the 1,2-addition reaction followed by hydrolysis to generate BCy-OH. Thus, the reason for the interaction between the spectroscopic probe and the Cys to release a significant optical signal was well elucidated. 

**Figure 2:**
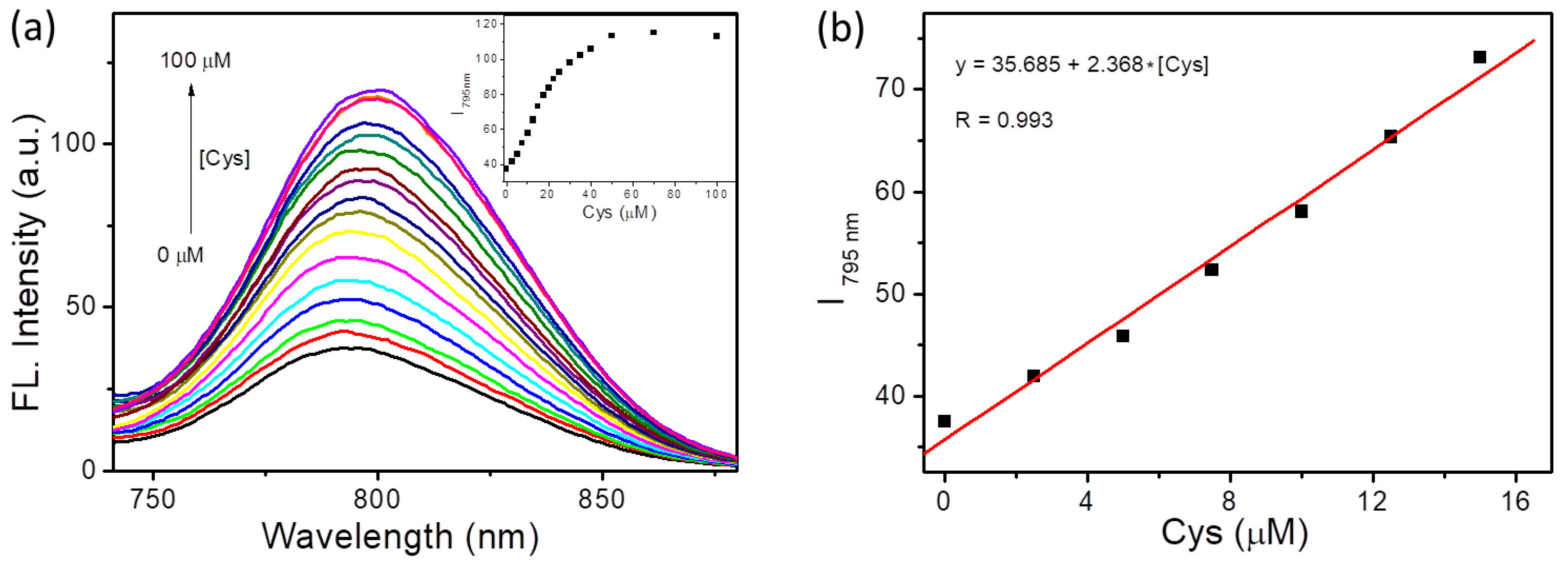
(**a**) The fluorescence spectral changes after 15 min of treatment with BCy-Cys (10 μM) versus a growing concentration of Cys (0–100 μM). Illustration: Scatter plot of emission intensity at 795 nm against various different levels of Cys. (**b**) The emission of BCy-Cys at 795 nm against various different levels of Cys (0–15 μM). The standard: PBS buffer solution (10 mM, pH 7.4, 25% ethanol), λ_ex_ = 720 nm, slit = 5/5 nm.

To further explore the sensitivity of BCy-Cys, concentration-dependent experiments were deployed. As depicted in Figure 2a, the emission wave at 795 nm gradually increased after adding different levels of Cys (0–100 μM), and the maximal emission spectral signals were observed in the presence of 5 equiv. of Cys. As well, similar variation currents were observed in the ultraviolet-visible absorption spectra. As depicted in Figure S5, after adding different levels of Cys (0–100 μM), the ultraviolet-visible absorption wave at 585 nm gradually disappeared and the ultraviolet-visible absorption wave at 750 nm gradually increased, eventually reaching the plateau when the quantity of Cys attained five equimolar. Moreover, by scrutinizing the emission spectra, we found that the emission intensity at the 795 nm wavelength tended to be linearly and directly related to the Cys level over a wide range. After calculation, the results showed that the emission intensity at 795 nm showed good linearity with the Cys levels (0–15 μM), and its correlation coefficient reached 0.993 (Figure 2b). The detection limit of BCy-Cys was calculated according to 3σ/k. Based on this formula, the limit of detection was estimated to be 0.31 μM, which is considerably lower than the physiological level of Cys in living cells (30–200 μM) [10], indicating that the sensitivity of BCy-Cys for the detection of Cys is very high (Figure 2b). In the above formula, k is the slope between emission intensity at 795 nm and Cys levels. The σ is the relatively normalized deviation of 11 parallel fluorescence emission intensity readings taken on a Cys-free blank solution. 

**Figure 3:**
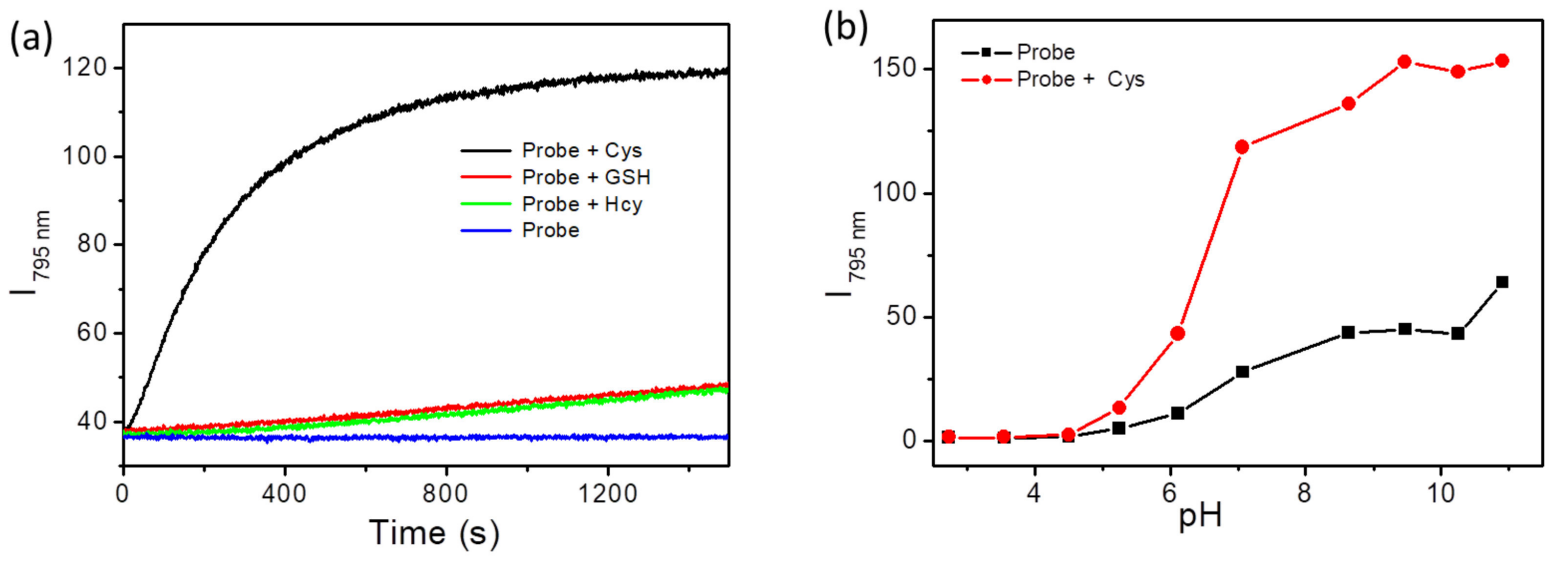
(**a**) Fluorescence emission intensity profiles at 795 nm of BCy-Cys (10 μM) as a function of time following exposure to Cys (black, 100 μM), Hcy (green, 100 μM), or GSH (bonus, 1 mM); (**b**) Emission intensity profiles at 795 nm of BCy-Cys (10 μM) with and without Cys (100 μM) attached with varied pH. The standard: PBS (10 mM, pH = 7.4, 25% ethanol), λ_ex_ = 720 nm, slit = 5/5 nm.

To further evaluate the sensing properties of BCy-Cys toward Cys, the time-dependent fluorescence change of BCy-Cys was deployed in the presence of Cys (100 μM). Meanwhile, in order to test the capability of the probe BCy-Cys to determine Cys over Hcy/GSH under cellular-relevant conditions, time-dependent fluorescence response experiments have been conducted upon incubation with high concentrations of GSH (1 mM) and Hcy (100 μM). As depicted in Figure 3a, by plotting the emission intensity of BCy-Cys at 795 nm with time, the fluorescence intensity rapidly rose after the incorporation of Cys and reached the plateau within about 15 min. In contrast, without the incorporation of Cys or upon addition of Hcy and GSH, respectively, the emission intensity of BCy-Cys at 795 nm remained substantially constant under the same condition, revealing that the background of the probe is extremely low and can distinguish Cys from Hcy/GSH within 15 min. The obtained data demonstrated that the probe BCy-Cys is capable of effectively differentiating Cys from Hcy/GSH under the same conditions or physiological conditions. Meanwhile, these results suggested that BCy-Cys had high photostability and could be employed as a good candidate for monitoring Cys in real-time.

In addition, pH is an important factor in the detection of substrates by fluorescent probes. Therefore, we further characterized the fluorescence response of BCy-Cys to Cys over a range of pH values (2.0–11.0) to extend the excellent functioning scope of the probe and enable the application of BCy-Cys to complex living functional systems. As depicted in Figure 3b (black line), the emission spectra of BCy-Cys exhibited relatively weak emission intensity at 795 nm and remained unaffected from 2.0 to 10.0. However, upon addition of Cys, the maximal emission signals were observed in the range of 6.0–12.0 (red line of Figure 3b). Cys is difficult to react with the probe, and the fluorescence signal is not obvious due to the reduced nucleophilicity of the sulfur atom of Cys in the acidic system (pH 2.0–5.0). However, with the increasing pH (6.0–11.0), the probe readily underwent a 1,2-addition reaction with Cys, which caused a dramatic enhancement of emission intensity at 795 nm. The broad tolerance of BCy-Cys to the pH at which Cys was detected and its stable fluorescence at a pH of around 7.4. The fluorescence test data presented above illustrates the potential of BCy-Cys for the detection of Cys in biological applications. 

**Figure 4:**
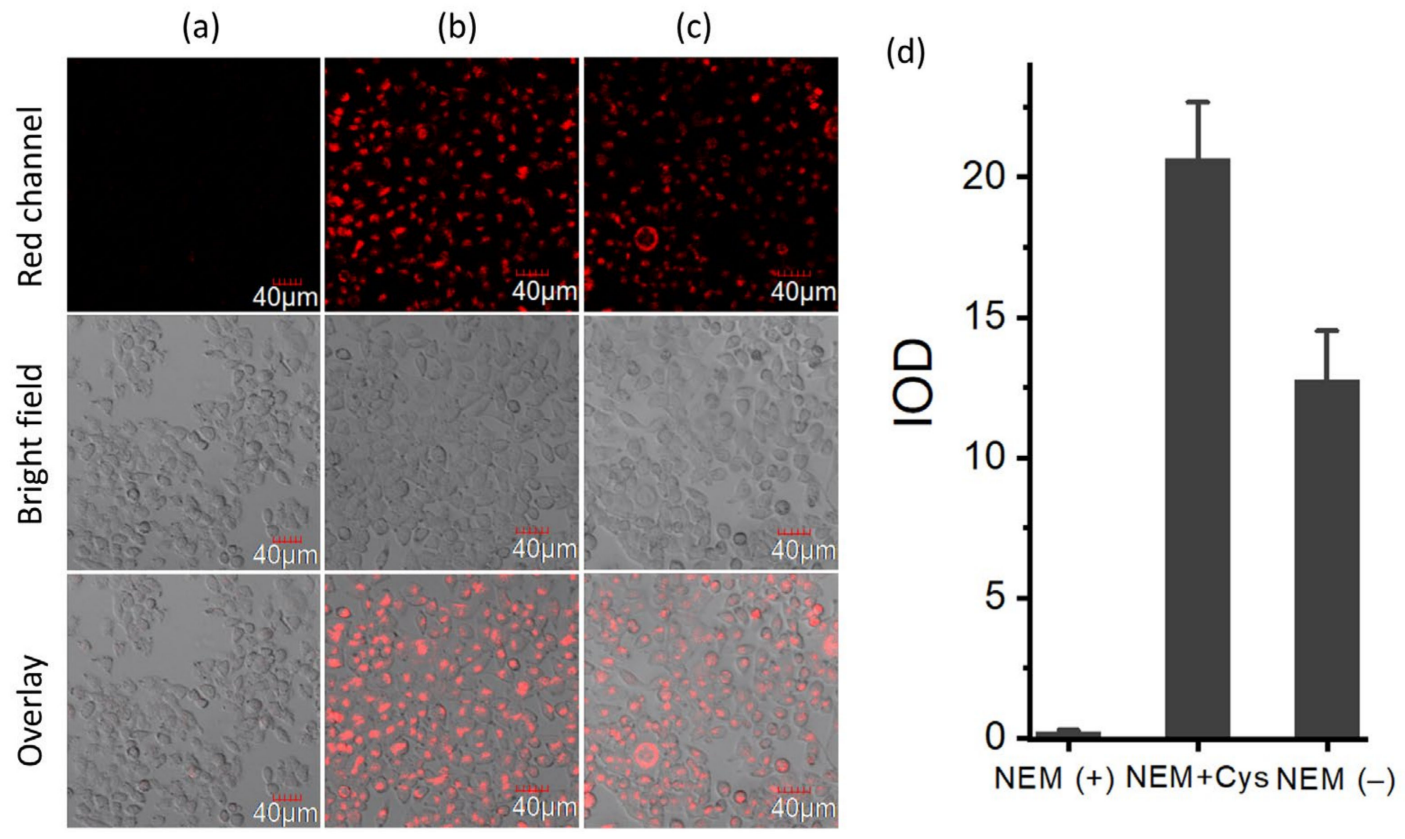
Laser confocal imaging pictures of HeLa cells. (**a**) HeLa cells were preincubated with NEM (1 mM) and colored by BCy-Cys (1 μM) for 30 min; (**b**) and inspected again using Cys (20 μM) for 30 min; (**c**) HeLa cells were inspected using BCy-Cys (1 μM) for 30 min; (**d**) optical density comparison. λ_ex_ = 635 nm, λ_em_ = 710–810 nm.

Motivated by the outstanding spectroscopic behavior of BCy-Cys in terms of great specificity, great agility, and physiological pH working scope, the promising application of this probe for Cys visualization in living cells was addressed in the following: Before the bioimaging assay, an MTT assay was first conducted to gauge the neurotoxicity of BCy-Cys within HeLa cells. As illustrated in Figure S6, cell survivability was high (not less than 80%) after treatment with varying levels of BCy-Cys (0, 0.1, 1, 5, 10, and 20 μM) over a period of 24 h. The higher cell survival reflected that the probe has low neurotoxicity to HeLa cells, which translated into high biosafety. The ability to be free from BCy-Cys neurotoxicity when conducting experimental imaging utilizing HeLa cells and BCy-Cys is very advantageous for experimental and biological applications. Subsequently, the capability of BCy-Cys to fluorescently image Cys with HeLa cells was examined. As illustrated in Figure 4a, the cells showed faint fluorescence signals across the red channel after *N*-ethylmaleimide (NEM, scavenging intracellular thiols) pretreatment, connecting the remarkably low native signal of the probe BCy-Cys. In contrast, a significant, strong luminescent signal across the red corridor was presented in the treated group (Figure 4b), and an about 100-fold fluorescence intensity higher than that of controlled cells was observed (Figure 4d), indicating that BCy-Cys possessed excellent cell membrane permeability and was responsive to Cys in living HeLa cells. Furthermore, when cells were cultivated with BCy-Cys for 30 min in the absence of NEM, a marked enhancement of the fluorescence color light signature of the red channel was noted (Figure 4c), with an enhancement of about 64-fold (Figure 4d), signifying that BCy-Cys could be suitable for the monitoring of the levels of endogenous Cys in biologically viable cells.

**Figure 5:**
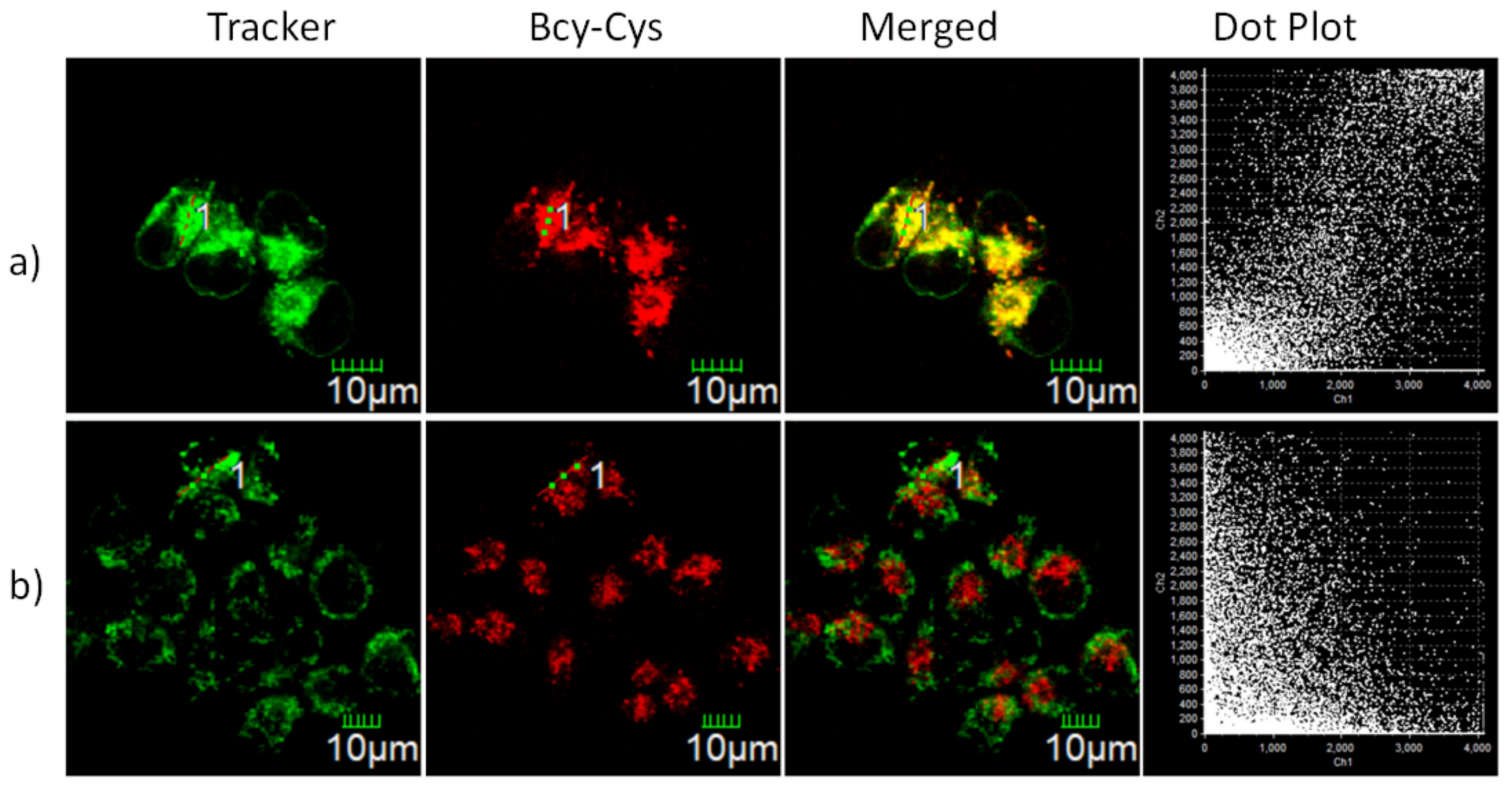
Fluorescence imaging of HeLa cells incubated with BCy-Cys (0.5 μM) and other organelle-targeting Trackers (100 nM) for 0.5 h. (**a**) Costained with Lyso-Tracker Green; (**b**) Costained with Mito-Tracker Green. 1: A linear region of interest across the cell. Red channel: λ_ex_ = 635 nm, λ_em_ = 710–810 nm. Green channel: λ_ex_ = 488 nm, λ_em_ = 511–539 nm.

Uneven fluorescence signals were identified in cells in Figure 4, intimating the accumulation of BCy-Cys in a particular organelle. Meanwhile, as we previously reviewed, thioheteroanthracene moieties or carbonyl selenium-functionalized xanthene group-functionalized xanthene moieties exhibited lysosomal targeting characteristics [24,25]. In order to test our hypothesis, the available common targeting assays Mito-Tracker Green and Lyso-Tracker Green were employed to characterize the organelle targeting of BCy-Cys and verify whether BCy-Cys has the desired lysosome localization ability. As illustrated in Figure 5a, we found that the red-colored signature from BCy-Cys overlapped well with the green fluorescence signals from Lyso-Tracker Green and showed yellow-colored pigments in the superimposed image with a Pearson’s coefficient as large as 0.90, indicating that BCy-Cys was dominantly partitioned in the lysosome. Nevertheless, the fluorescent-colored signatures of the probe and Mito-tracker Green were clearly located in different regions (Figure 5b). Meanwhile, the Pearson’s coefficient (0.25) is lower in the scatterplot for the intensities plotted for the green and red color passages. Co-localization experiments confirmed that BCy-Cys could be used to monitor endogenous Cys in the lysosome.

## 3. Materials and Methods

### 3.1. Synthesis

The key compound 2-(2-(2-chloro-3-(-2-(3-ethylbenzo[d]thiazol-2(3H)-ylidene) ethylidene)cyclohex-1-en-1-yl)vinyl)-3-ethylbenzo[d]thiazol-3-ium (BCy) was synthesized according to the procedures reported [24].

Synthesis of (*E*)-3-ethyl-2-(2-(6-hydroxy-2,3-dihydro-1H-thioxanthen-4-yl)vinyl)benzo[d]thiazol-3-ium (BCy-OH): After dissolving 3-hydroxythiophenol (200 μL, 2 mmol) in acetonitrile (8 mL), K_2_CO_3_ (276 mg, 2 mmol) was added under an Ar atmosphere and stirred at room temperature for 10 min. Furthermore, a suspension of BCy (579 mg, 1 mmol) in acetonitrile (5 mL) was pipetted slowly. After cooling to room temperature, the residue was filtered off, and the filtrate evaporated. The product was separated by column chromatography to give 300 mg of a dark blue powdered solid (61% yield). M.p. 210–212 °C; ^1^H NMR (400 MHz, DMSO-*d_6_*, ppm): *δ* 8.27 (d, *J* = 8.0 Hz, 1H), 8.12 (d, *J* = 8.4 Hz, 1H), 8.00 (d, *J* = 14.0 Hz, 1H), 7.78 (t, *J* = 7.8 Hz, 1H), 7.67 (t, *J* = 7.8 Hz, 1H), 7.39 (d, *J* = 8.4 Hz, 1H), 7.09 (d, *J* = 11.6 Hz, 1H), 6.91 (s, 1H), 6.80 (d, *J* = 8.8 Hz, 2H), 4.80–4.74 (m, 2H), 2.65 (d, *J* = 4.8 Hz, 4H), 1.81 (t, *J* = 5.2 Hz, 2H), 1.40 (t, *J* = 6.8 Hz, 3H); ^13^C NMR (100 MHz, DMSO-*d_6_*, ppm): *δ* 170.1, 159.2, 146.9, 142.6, 141.6, 134.1, 133.3, 132.5, 130.7, 129.6, 128.0, 127.8, 125.6, 124.5, 121.7, 116.5, 116.2, 111.0, 108.4, 43.8, 32.1, 27.4, 20.6, 14.1; HRMS *m/z* = 404.1143, calcd for C_24_H_22_NOS_2_^+^ [M]^+^, found: 404.1154.

Synthesis of (*E*)-2-(2-(6-(acryloyloxy)-2,3-dihydro-1H-thioxanthen-4-yl)vinyl)-3-ethylbenzo[d]thiazol-3-ium (BCy-Cys): After dissolving the product BCy-OH (100 mg, 0.2 mmol) obtained in the previous step and drying Et_3_N (0.4 mmol, 60 µL) in dichloromethane, acryloyl chloride (0.4 mmol, 32 µL) was gradually added in an ice bath under argon ambient, followed by vigorous stirring at ambient temperature for reaction. After removal of the solvent by means of a rotary evaporator, the crude material was clarified on a silica gel column to give the bluish BCy-Cys chemical composition (20 mg, 20% yield). M.p. 200–202 °C; ^1^H NMR (400 MHz, DMSO-*d_6_*): δ 8.35–8.33 (d, *J* = 8.0 Hz, 1H), 8.21–8.19 (d, *J* = 8.4 Hz, 1H), 8.01–7.97 (d, *J* = 14.8 Hz, 1H), 7.84–7.81 (t, *J* = 7.8 Hz, 1H), 7.75–7.71 (t, *J* = 7.8 Hz, 1H), 7.57–7.55 (d, *J* = 8.4 Hz, 1H), 7.47–7.46 (d, *J* = 2.0 Hz, 1H), 7.27–7.24 (d, *J* = 14.4 Hz 1H), 7.20–7.18 (m, 1H), 7.09 (s, 1H), 6.59–6.54 (m, 1H), 6.46–6.40 (m,1H), 6.22–6.19 (m, 1H), 4.86–4.81 (m, 2H), 2.72–2.67 (m, 4H), 1.86–1.83 (t, *J* = 5.6 Hz, 2H), 1.43–1.40 (t, *J* = 7.0 Hz, 3H); ^13^C NMR (100 MHz, DMSO-*d_6_*): δ 170.7, 164.3, 150.7, 143.9, 142.6, 141.6, 134.8, 134.1, 132.8, 131.4, 130.8, 129.9, 128.4, 128.2, 127.8, 127.6, 127.3, 124.7, 121.8, 118.5, 116.6, 110.8, 44.3, 32.2, 29.5, 27.6, 22.6, 20.5, 14.3; HRMS *m/z* = 458.1248 calcd for C_27_H_24_NO_2_S_2_^+^ [M]^+^, found: 458.1221.

### 3.2. MTT Assay

The MTT assay was undertaken in accordance with our earlier work [24,25].

### 3.3. Cell Culture and Fluorescence Imaging

HeLa cells were cultivated in accordance with our earlier reports [25,26]. The cells were previously prefluidized in or out of NEM (1 mM) for 30 min and then colored with 1 μM BCy-Cys for 30 min. In the other group, the cells were previously prefluidized with BCy-Cys (1 μM) and then cultivated with Cys (100 μM) for 30 min in PBS buffer (10 mM, pH = 7.4). For co-localization experiments, 100 nM of common targeting trackers (Mito-Tracker Green and Lyso-Tracker Green) and BCy-Cys (0.5 μM) were cultivated for 30 min, respectively. The media was rinsed three times with PBS buffer before immortalizing the cells, employing laser confocal microscopy to remove excess probes for better imaging.

## 4. Conclusions

Collectively, we provided a promising lysosomally aimed NIR fluorescent probe for selective assay of Cys over Hcy/GSH by modifying the thioxanthene-benzothiozolium fluorophore. Unsurprisingly, BCy-Cys was not only able to demonstrate the emission wavelengths in the NIR region but also nondestructively detect Cys within the lysosome, better realizing our design intent. The probe is capable of emitting at a wavelength of 795 nm after Cys treatment, which is a very high wavelength. More specifically, BCy-Cys exhibited high selectivity and high sensitivity for the rapid detection of Cys over Hcy/GSH, with an extremely low limit of detection at 0.31 μM. Furthermore, it is also noteworthy that the color of the solution turned from bright blue to cyan in the presence of Cys, suggesting that BCy-Cys can be used for ‘naked eye’ determination of Cys. Importantly, BCy-Cys could be used for imaging exogenous and endogenous lysosome Cys, indicating its potential applications in complex biological systems.

## Data Availability

Not applicable.

## Supplementary Materials

The following supporting information can be downloaded at: https://www.mdpi.com/article/10.3390/molecules28176189/s1. Figure S1: Color change of BCy-Cys; Figure S2: Fluorescence intensity of BCy-Cys at 795 nm upon addition of different species in the absence/presence of Cys; Figure S3: HRMS spectrum of BCy-Cys recorded after reaction with Cys; Figure S4: Absorption and fluorescence emission responses of BCy-OH and BCy-Cys upon addition of Cys; Figure S5: Changes in the absorption spectra of BCy-Cys at growing Cys concentrations; Figure S6: MTT assay for the survival rate of HeLa cells treated with various concentrations of BCy-Cys; Figures S7–S12: ^1^H, ^13^C NMR spectra and HRMS of BCy-OH and BCy-Cys. Table S1: Comparison of BCy-Cys with known probes [27,28,29,30,31,32,33,34,35,36].

## Sample Availability

Samples of the compounds BCy-OH and BCy-Cys are available from the authors.
