# Supplementary material for: A New Lysosome-Targeted NIR Fluorescent Probe for Specific Detection of Cysteine over Homocysteine and Glutathione
Source: Molecules. 2023 Aug 22;28(17):6189. doi: 10.3390/molecules28176189 (PMC10489057; doi:10.3390/molecules28176189)
Supplement: Supplementary file 1 [file molecules-28-06189-s001.zip › molecules-2543832-supplementary.pdf]

Supporting Information

A New Lysosome-Targeted NIR Fluorescent Probe for Specific Detection of Cysteine over Homocysteine and Glutathione

Qiuchen Liu [1,2,3], Chang Liu [2], Song He [2], Xianshun Zeng [2], Jian Zhang [1,*] and Jin Gong [1,2,*]

1 School of Pharmacy, Weifang Medical University, Weifang 261053, China
2 Tianjin Key Laboratory for Photoelectric Materials and Devices, School of Materials Science & Engineering, Tianjin University of Technology, Tianjin 300384, China
3 School of Chemical Engineering and Technology, Tianjin University, Tianjin 300072, China
* Correspondence: zhangjian_3323@163.com (J.Z.); gongjin@wfmc.edu.cn (J.G.)

Contents

Materials and instruments..S2

The comparison of **BCy-Cys** with known probes..S2-S3

Color change of **BCy-Cys**..S4

Interference experiment..S4

HRMS of **BCy-Cys** reaction with Cys..S4

Absorption and emission responses of **BCy-OH** and **BCy-Cys** upon addition of Cys..S5

The absorption changes of **BCy-Cys** treated with increasing concentrations of Cys..S5

MTT assay..S5

^{1}H, ^{13}C NMR spectra and HRMS of **BCy-OH and BCy-Cys**............................S6-S8

Materials and instruments

The various reagents and organic solvents used in the experiments were of analytical grade and were purchased from J&K Scientific Ltd. All reagents and organic solvents required no further purification and were used directly unless otherwise stated. The water used in the experiments was double distilled water. The progress of the reaction was monitored by observing a thin layer chromatography under an ultraviolet lamp. The thin layer chromatography used in the experiments and the silica gel (200-400 mesh) used in the column chromatography were purchased from Qingdao Ocean Chemicals. Absorption spectra were measured on UV-2550 UV/Vis spectrophotometer (Hitachi Japan), and fluorescence emission spectra were measured on F-4600 fluorescence spectrophotometer (Hitachi Japan). All fluorescence spectra data were at an excitation wavelength of 500 nm with an excitation/emission slit width of 10/10 nm. ^{1}H NMR (400 MHz) and ^{13}C NMR (100 MHz) spectra were collected by Bruker spectrometer. Chemical shift (δ) values are in ppm and tetramethylsilane is used as an internal standard. High resolution

mass spectra (HRMS) were measured by Agilent 6510 Q-TOF LC/MS instrument (Agilent Technologies, Palo Alto, CA) equipped with an electrospray ionization (ESI) source. The pH was measured using a FE 20/EL 20PH meter (Mettler-Toledo Instruments (Shanghai) CO., Ltd.). Cell imaging was performed by Olympus FV 1000-IX81 laser scanning confocal imaging. All images were analyzed with Olympus FV1000-ASW. The cells used in the manuscript were purchased from Beijing Dingguo Changsheng Biotechnology Co., Ltd.

Table S1. The comparison of BCy-Cys with known probes [27–36].

**No.**	Structures	λ_{em} (nm)	LOD (nM)	Time (min)	**Selectivity (Cys, Hcy, GSH)**	**Ref.**
1	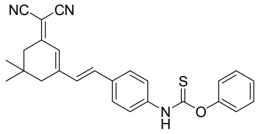	660	79	60	good	[27]
2	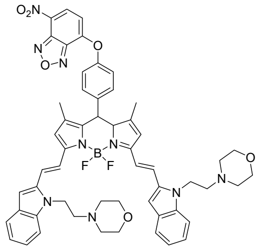	735	22	30	poor	[28]
3	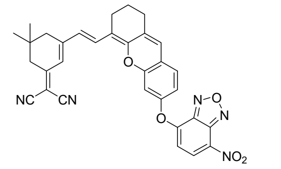	756	1010	8	poor	[29]
4	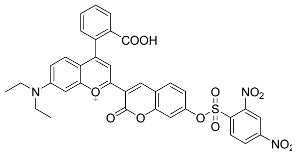	564	17.3	20	poor	[30]
5	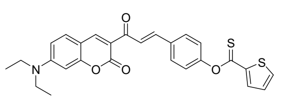	556	240	10	poor	[31]
6	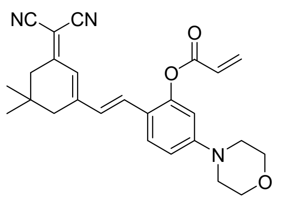	670	35.2	5	poor	[32]
7	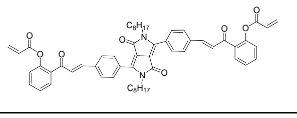	605	290	6	poor	[33]

8	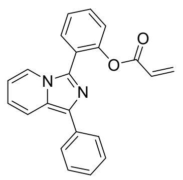	475	70	10	good	[34]
9	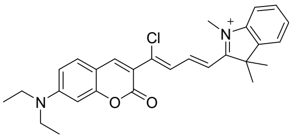	725	2965	60	poor	[35]
10	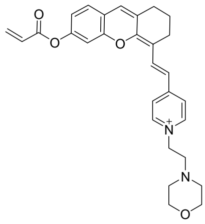	674	960	-	good	[36]
11	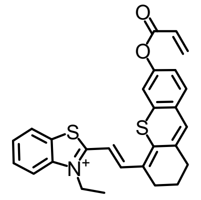	**795**	**310**	**15**	**good**	**This work**

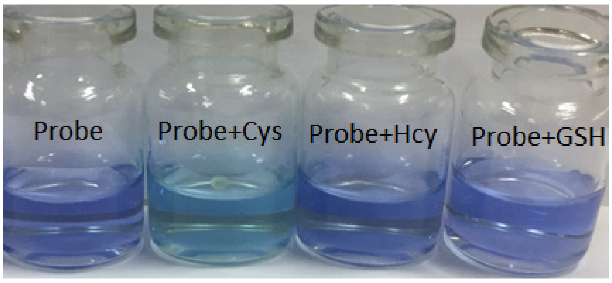

Figure S1. Color change of **BCy-Cys** (10 μM) before and after the addition of Cys (100 μM), Hcy (100 μM), and GSH (1 mM).

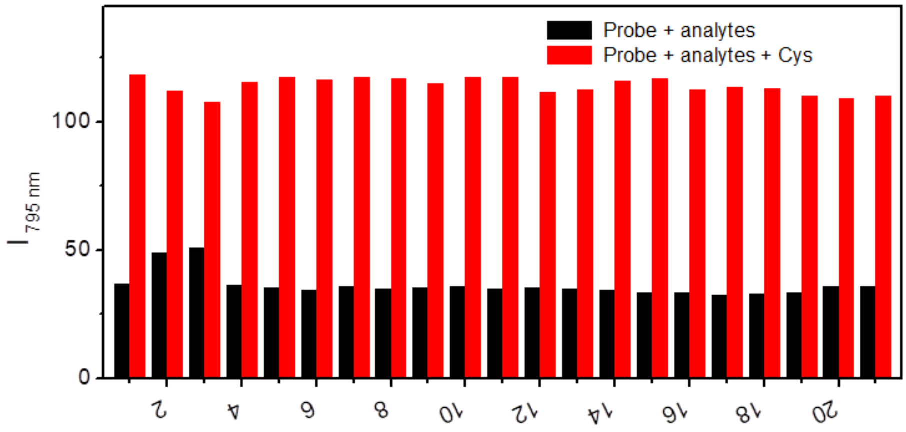

Figure S2. Fluorescence intensity of **BCy-Cys** (10 μM) at 795 nm upon addition of different species (100 μM) in the absence/presence of Cys (100 μM). 1-21: blank, Hcy, GSH (1 mM), Phe, Ala, Met, Glu, Arg, Lys, Tyr, Leu, Pro, Trp, Ser, Thr, Val, His, Na^{+} (1 mM), K^{+} (1 mM), Ca^{2+} (1 mM), and Mg^{2+} (1 mM). The conditions: PBS/Ethanol (3:1, v/v), λ_{ex} = 720 nm, slit = 5/5 nm.

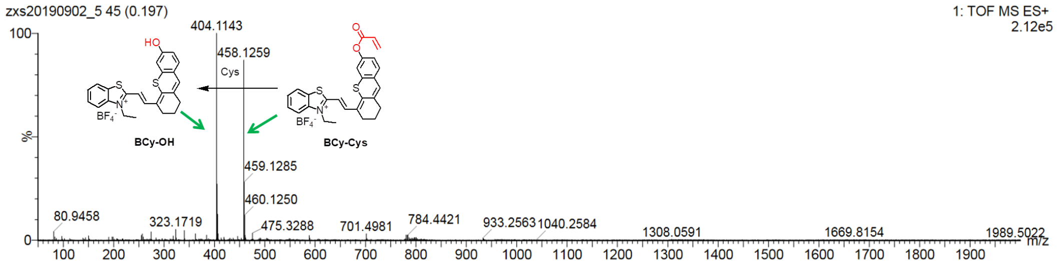

Figure S3. HRMS spectrum of **BCy-Cys** recorded after reaction with Cys.

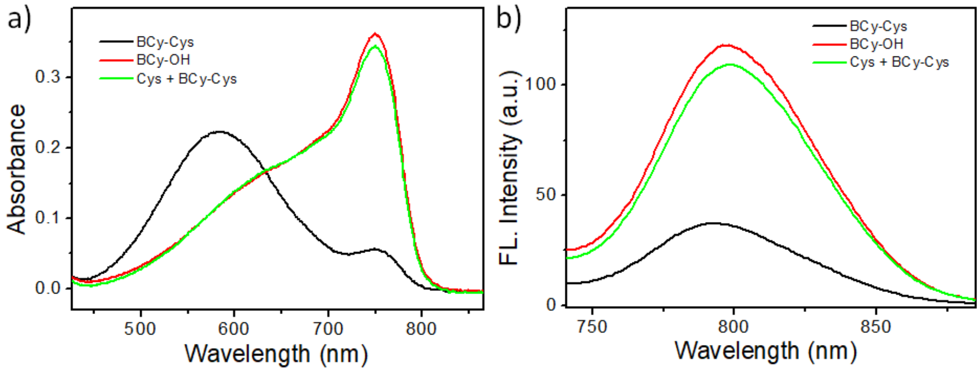

Figure S4. a) Absorption, and b) fluorescence emission responses of **BCy-OH** (10 μM) and **BCy-Cys** (10 μM) upon addition of Cys (100 μM). The conditions: PBS/Ethanol (3:1, v/v), λ_{ex} = 720 nm, slit = 5/5 nm.

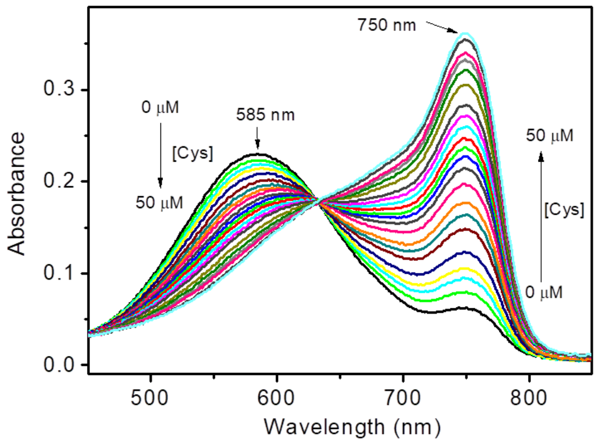

Figure S5. The absorption changes of **BCy-Cys** (10 μM) treated with increasing concentrations of Cys (0 – 50 μM).

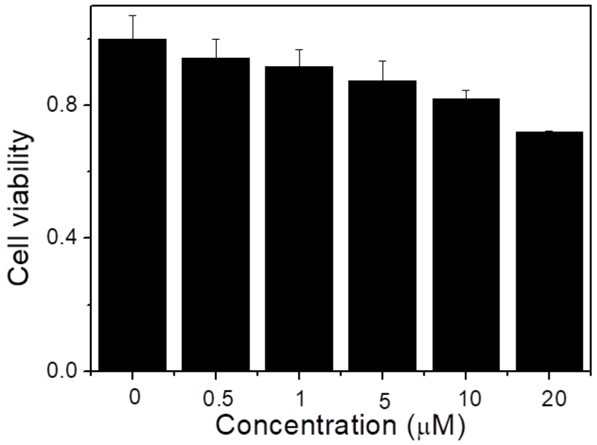

Figure S6 MTT assay for the survival rate of HeLa cells treated with various concentrations of **BCy-Cys** for 24 h. Error bars represent the standard deviations of 5 trials.

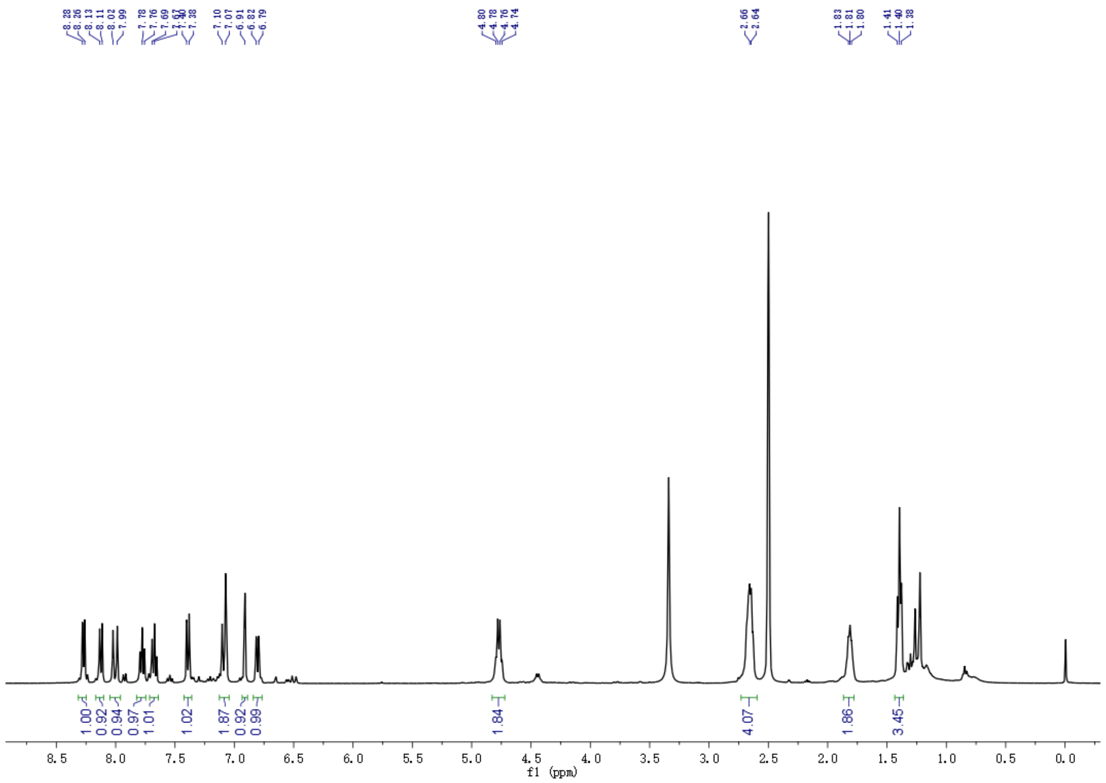

Figure S7 ^{1}H NMR spectra of compound **BCy-OH** in DMSO-d_6.

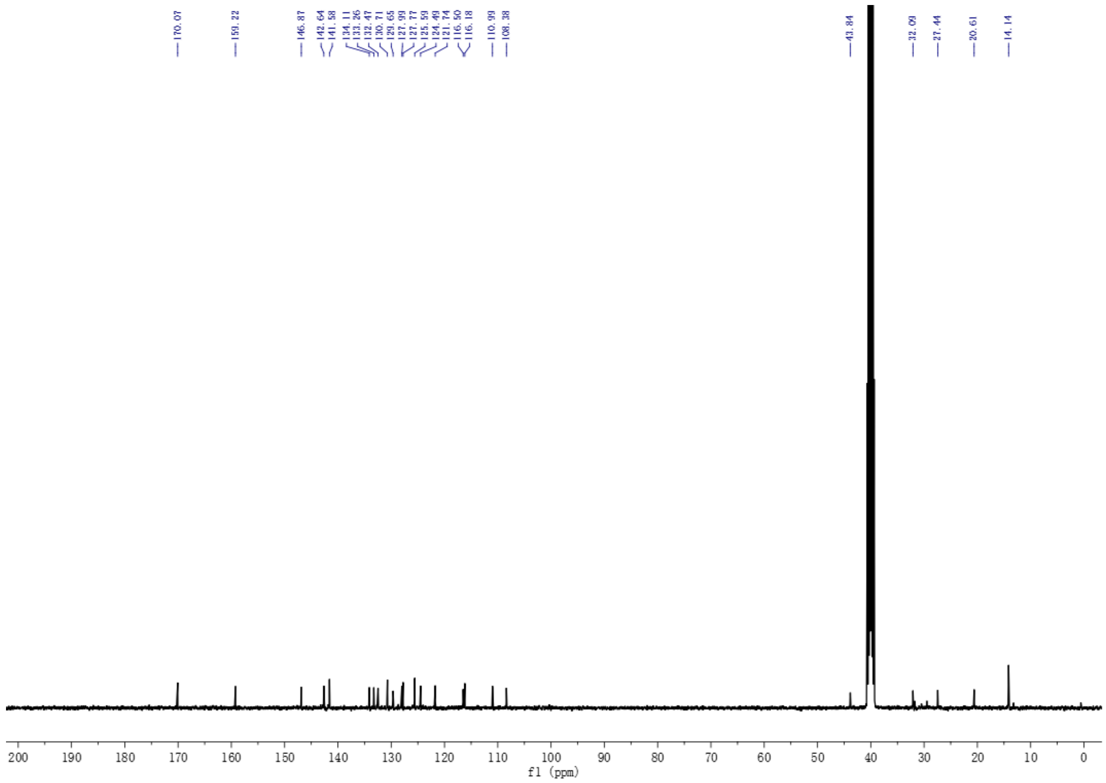

Figure S8 ^{13}C NMR spectra of compound **BCy-OH** in DMSO-d_6.

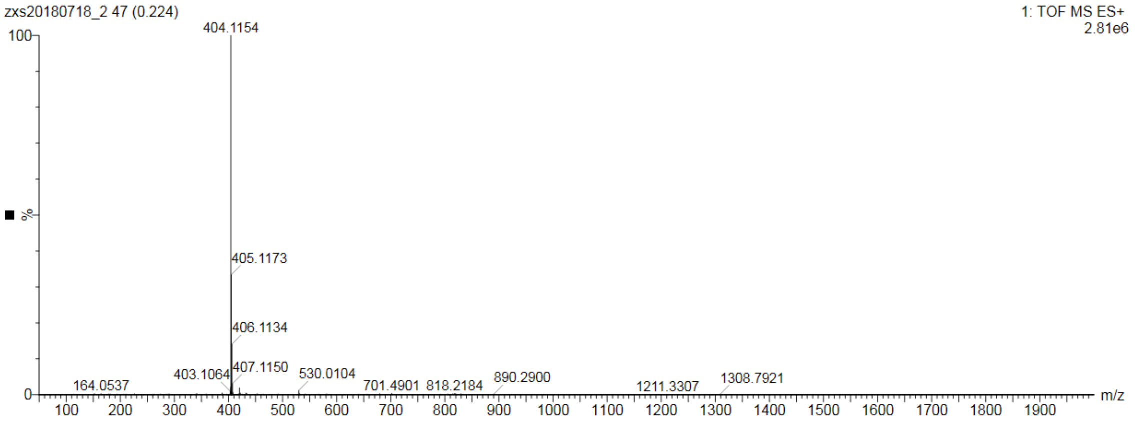

Figure S9. HRMS spectrum of compound **BCy-OH**.

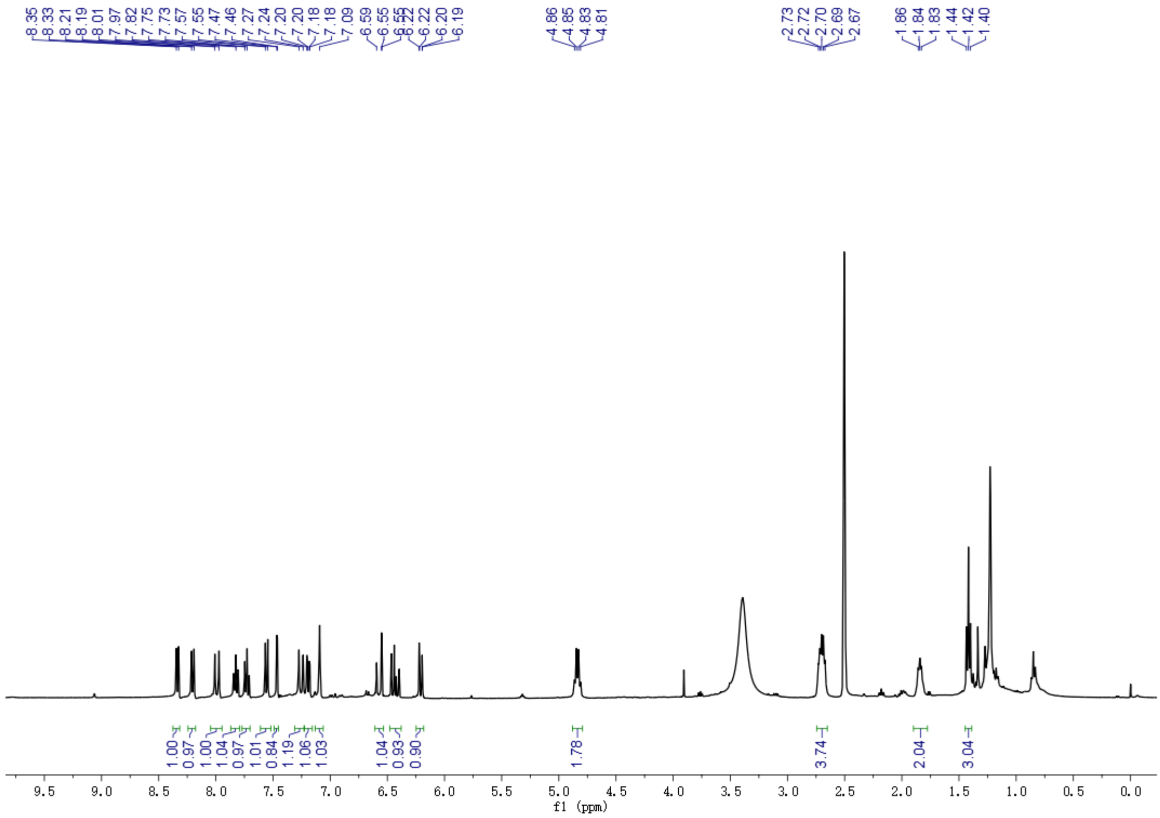

Figure S10 ^{1}H NMR spectra of compound **BCy-Cys** in DMSO-d_6.

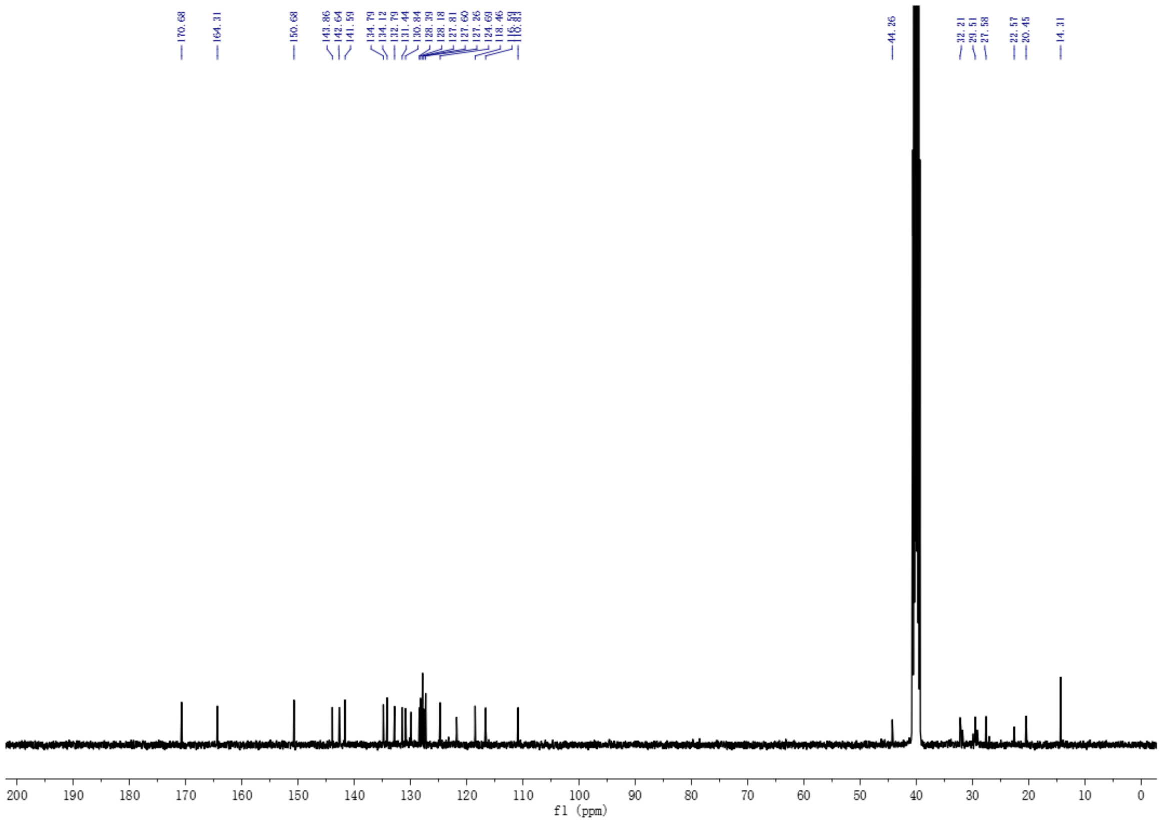

Figure S11 ^{13}C NMR spectra of compound **BCy-Cys** in DMSO-d_6.

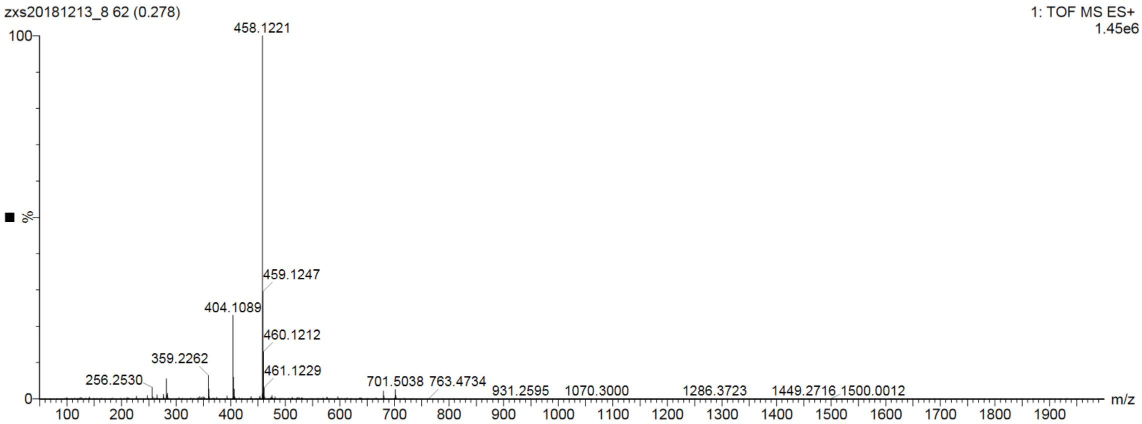

Figure S12 HRMS spectra of compound **BCy-Cys**.
